# Empirical evaluation of internal validation methods for prediction in large-scale clinical data with rare-event outcomes: a case study in suicide risk prediction

**DOI:** 10.1186/s12874-023-01844-5

**Published:** 2023-02-01

**Authors:** R Yates Coley, Qinqing Liao, Noah Simon, Susan M. Shortreed

**Affiliations:** 1 Kaiser Permanente Washington Health Research Institute, 1730 Minor Ave. #1600, Seattle, WA 98101 USA; 2 Department of Biostatistics, University of Washington, Seattle, WA USA

**Keywords:** Bootstrap, Clinical prediction, Cross-validation, Machine learning, Optimism, Random forest, Risk stratification, Split-sample

## Abstract

**Background:**

There is increasing interest in clinical prediction models for rare outcomes such as suicide, psychiatric hospitalizations, and opioid overdose. Accurate model validation is needed to guide model selection and decisions about whether and how prediction models should be used. Split-sample estimation and validation of clinical prediction models, in which data are divided into training and testing sets, may reduce predictive accuracy and precision of validation. Using all data for estimation and validation increases sample size for both procedures, but validation must account for overfitting, or *optimism*. Our study compared split-sample and entire-sample methods for estimating and validating a suicide prediction model.

**Methods:**

We compared performance of random forest models estimated in a sample of 9,610,318 mental health visits (“entire-sample”) and in a 50% subset (“split-sample”) as evaluated in a prospective validation sample of 3,754,137 visits. We assessed optimism of three internal validation approaches: for the split-sample prediction model, validation in the held-out testing set and, for the entire-sample model, cross-validation and bootstrap optimism correction.

**Results:**

The split-sample and entire-sample prediction models showed similar prospective performance; the area under the curve, AUC, and 95% confidence interval was 0.81 (0.77–0.85) for both. Performance estimates evaluated in the testing set for the split-sample model (AUC = 0.85 [0.82–0.87]) and via cross-validation for the entire-sample model (AUC = 0.83 [0.81–0.85]) accurately reflected prospective performance. Validation of the entire-sample model with bootstrap optimism correction overestimated prospective performance (AUC = 0.88 [0.86–0.89]). Measures of classification accuracy, including sensitivity and positive predictive value at the 99^th^, 95^th^, 90^th^, and 75^th^ percentiles of the risk score distribution, indicated similar conclusions: bootstrap optimism correction overestimated classification accuracy in the prospective validation set.

**Conclusions:**

While previous literature demonstrated the validity of bootstrap optimism correction for parametric models in small samples, this approach did not accurately validate performance of a rare-event prediction model estimated with random forests in a large clinical dataset. Cross-validation of prediction models estimated with all available data provides accurate independent validation while maximizing sample size.

**Supplementary Information:**

The online version contains supplementary material available at 10.1186/s12874-023-01844-5.

## Background

There is growing interest in using prediction models to guide clinical care [1]. A key aspect of developing a clinical prediction model intended is validation, or assessing performance for observations outside of the original data used for model estimation [2]. *Internal validation* refers specifically to methods that use the same data available for model estimation to anticipate performance in new observations from the same underlying sample [3]. Accurate internal validation is important to correctly guide both model selection (choosing an optimal model among several candidate prediction models) and decisions about whether and how a prediction model should be used in clinical practice.

We considered the use-case of a prediction model to estimate suicide risk in the 90 days following an outpatient mental health visit. Mental health providers are particularly interested in identifying patients at high risk of suicide at clinical encounters so that suicide prevention interventions can be made. In our dataset of over 13 million visits, suicide is a rare event—there are 23 events per 100,000 visits. The data also contain over a hundred predictors characterizing patient’s clinical history, including mental health diagnoses, medications, and encounters. In this empirical evaluation, our goal was two-fold: first, to estimate the best possible suicide prediction model to optimize identification of high-risk patients and, second, to accurately estimate how well the prediction model will perform in future use.

Split-sample estimation and validation, in which the entire available sample is randomly divided into two subsets, one used exclusively for model estimation (“training”) and another used for validation (“testing”), is common [4]. Using an independent validation set avoids overestimating model performance, [5] as occurs when performance is evaluated in the same sample used to the construct the model [6]. However, using only a fraction of available observations exclusively for estimation and the remainder exclusively for validation reduces the statistical power of both tasks [7]. Using a smaller sample for training increases the risk that variable selection methods will miss an important predictor and estimated models will have greater variability [8]. Using a smaller sample for validation results in less precise estimates of performance [9]. Sample size limitations for estimation and validation are particularly important to consider when predicting a rare event, such as suicide.

Methods have been proposed that use the entire available dataset for both model estimation and validation. Using all available data for estimation requires validation methods that account for the potential for overfitting, that is, when a prediction model is accurate in the sample used for estimation but not accurate for new observations. Overfitting causes *optimism*, or over-estimation of performance, when performance is assessed in the same data used for estimation. Optimism is likely to be larger when the number of events is small relative to the number of predictors and when using more flexible prediction models [10, 11].

In this study, we considered two validation methods to adjust for optimism error when using the same sample for both prediction model estimation and validation: cross-validation and bootstrap optimism correction. Cross-validation [12] is typically employed to guide tuning parameter selection during the estimation process, and out-of-fold predictions can also be used for model validation. In smaller samples or when the event is rare, performance estimates from each fold can be highly variable [13, 14], so cross-validation may need to be repeated (for e.g., 5 × 5-fold cross-validation where 5-fold cross-validation is repeated on 5 different splits of the dataset) to obtain stable estimates of model performance [13, 14].

Alternatively, some authors have advocated that applying an optimism correction estimated via bootstrap sampling is the preferred method to obtain unbiased and efficient estimates of the predictive performance of a model estimated in the entire sample [9, 15–17]. However, demonstrations of bootstrap optimism correction for continuous risk prediction have been limited to logistic regression models predicting relatively common events (prevalence at or above 1%) in small samples with a handful of predictors selected by stepwise methods [9, 18]. In this context, as sample size increases, both the risk of overfitting and relative efficiency gain from using bootstrap optimism correction over cross-validation and split-sample validation decline [19]. These settings do not reflect the current context of clinical prediction modeling in which a wide range of machine learning methods are used to estimate prediction models with hundreds, or even thousands, of predictors extracted from health records data on millions of patients [20].

Using the entire sample for both model estimation and validation is particularly appealing for predicting rare events to address concerns about missing important predictors, uncertainty in model estimation, and high variability in performance estimates that arise even in samples with millions of observations. While estimating a prediction model with the entire sample may result in better clinical performance, accurate internal validation that accounts for overfitting remains a concern; it is unknown whether optimism estimates obtained via bootstrap accurately correct for optimism when prediction models are constructed with data-driven (or “data-hungry”) [21] machine learning techniques with many predictors and a rare event.

We empirically evaluated spit-sample and entire-sample methods for estimating and validating a random forest prediction model for risk of suicide following an outpatient mental health visit. Our analysis investigated two questions. First, we examined whether a suicide prediction model estimated in the entire sample, instead of a subset of data, improved risk prediction as evaluated in a prospective validation set. Second, we assessed whether three internal validation approaches adequately adjusted for overfitting to produce accurate performance estimates by comparing validation estimates from each approach—(1) validation in an independent testing set of the prediction model estimated with the split-sample approach and, for the prediction model estimated in the entire sample, (2) cross-validation and (3) bootstrap optimism correction —to prospective performance.

## Methods

### Data

Our sample included all outpatient mental health specialty visits for patients age 13 years or older at seven health systems (HealthPartners; Henry Ford Health System; and the Colorado, Hawaii, Northwest, Southern California, and Washington regions of Kaiser Permanente) between January 1, 2009 and September 30, 2017 (or the latest date cause of death data were available, Table S1). The predicted outcome was suicide within 90 days following the visit. Suicides were identified from state mortality records indicating definite or probably suicide [22, 23]. A person who died by suicide may have had multiple visits in the 90 days preceding suicide; thus, a single suicide may be attributed to more than one visit in the sample.

Predictors of suicide were extracted from clinical records and insurance claims data. Predictors included demographics (age, sex, race, ethnicity, insurance type); 3-month, 1-year, and 5-year history of mental health and substance use diagnoses, prescriptions, and encounters (inpatient, outpatient, and emergency department); comorbidities (measured by Charlson Comorbidity Index categories); [24] prior suicide attempts; and responses to the 9-item Patient Health Questionnaire (PHQ-9), [25] a patient-reported measure of depressive symptoms, including thoughts of suicide and self-harm (Table S2). Calendar time is not included as a predictor of suicide risk, as this prediction model is intended for prospective use in clinical care. Additional information about data collection methods can be found in Simon et al. 2018 [26] and at www.github.com/MHResearchNetwork.

The sample was divided into a *development dataset* containing all visits from January 1, 2009-September 30, 2014 and a *prospective validation dataset* containing visits from January 1, 2015- September 30, 2017. The development dataset was used for prediction model estimation and internal validation. The prospective validation dataset was used to evaluate future performance of prediction models estimated in the development dataset; we used this set-up to reflect predictive performance if models were applied in the same population and setting later in time [2].

### Prediction model estimation and validation

First, we describe the general methodology we used to estimate random forest models with our dataset. We then describe the different approaches we evaluated for estimating prediction models and validating performance.

#### Random forest prediction models

We used random forest models to estimate the probability of 90-day suicide [27]. Random forests have two hyperparameters that we selected for estimation: the minimum terminal node size, which dictates how deep each tree grows, and the number of predictors randomly selected for consideration at each split. Optimal minimum terminal node size depends on sample size, so we considered different parameter values for the split-sample training set (10,000, 25,000, 50,000, 100,000, and 150,000 visits) and for model estimation with the entire sample (50,000, 100,000, 250,000, and 500,000 visits). For number of predictors selected at each split, we considered 11 predictors; this is the square root of the total number of predictors in the dataset (rounded down), the recommended default for classification trees [11]. We also considered 22 and 5 predictors, twice and half as many as the recommended default, respectively. With each sample used for model estimation, 5-fold cross-validation was used to select the tuning parameter combination that optimized out-of-fold area under the curve (AUC), Table S3 [28]. Additional specifications of random forest prediction models are described in supplementary materials.

#### Evaluation of prediction model performance

We selected measures of prediction model performance that reflected how our health system partners plan to use suicide prediction models to inform mental health care. From our experience, health system leaders choose a threshold of the continuous risk score to classify patients as “high-risk” (i.e., risk score at or above the threshold). Suicide prevention interventions are then offered at visits classified as high-risk. To evaluate possible thresholds, health system stakeholders need to know whether each binary categorization of risk scores identifies people in need of suicide prevention (sensitivity) while limiting unnecessary interventions (specificity). At each threshold, the intensity and cost of suicide prevention interventions must also be balanced against the event rate in those flagged as high-risk (positive predictive value [PPV]). Thresholds are selected based on classification accuracy as well as health system capacity and intervention effectiveness.

To inform health system decisions about whether and how to implement a suicide prediction model, we focus prediction model evaluation on measures of classification accuracy (sensitivity, specificity, and PPV) at thresholds under consideration—the 99^th^, 95^th^, 90^th^, and 75^th^ percentiles of the suicide risk score distribution. (Negative predictive value [NPV] was not included in our presentation; because suicide is a rare event, NPV is nearly one at all percentiles examined). Prediction model performance was also evaluated using area under the curve (AUC), as this measure summarizes sensitivity and specificity across the range of all possible thresholds.

#### Split-sample prediction model estimation and internal validation

For the split-sample approach, we divided the development dataset into a *training set* containing all visits from a random sample of half of the people in the dataset. All visits from the remaining 50% comprised the *testing set* used for internal validation. 5-fold cross-validation was done in the training set to identify the tuning parameter combination that optimized out-of-fold AUC. After tuning parameters were selected, the final split-sample prediction model was estimated with all visits in the training set.

The split-sample prediction model was then applied to obtain suicide risk predictions for all visits in the testing set. AUC and classification accuracy of the split-sample prediction model was evaluated in the testing set Classification accuracy was calculated for thresholds defined at percentiles of the suicide risk prediction distribution in the training set (that is, they were determined independently of the distribution of predictions in the testing set). We constructed 95% confidence intervals (CIs) of performance measures in the testing set via 1,000 bootstrap samples [16]. Bootstrap resampling was done at the person-level [29] and stratified by event status (person with suicide following any visit vs. person without suicide).

#### Entire-sample prediction model estimation and internal validation

All visits in the development dataset were used for the entire-sample prediction model estimation and internal validation. 5-fold cross-validation within the entire development dataset was used to identify the tuning parameter combination that optimized out-of-fold AUC. The final entire-sample prediction model was estimated using all visits in the development sample and the selected tuning parameters.

#### Cross-validation for internal validation

Cross-validation was used to estimate entire-sample model performance. Following cross-fold validation to select the optimal tuning parameters for the entire-sample prediction model, out-of-fold predictions for chosen hyperparameters were saved. Performance metrics were calculated in the out-of-fold predictions. For measures of classification accuracy, prediction thresholds were defined in the distribution of in-sample predictions in the entire development sample. Quantile-based 95% CIs around performance measures were estimated in 500 event-stratified person-level bootstrap samples. (Bootstrapping resampling was only repeated 500 times for both entire-sample validation methods to reduce computational burden. Moving estimates of the mean and 95% CI were monitored to ensure estimate stability, Figs. S1, S2).

#### Bootstrap optimism correction for internal validation

Bootstrap sampling was also used to produce optimism-corrected estimates of all validation metrics. [9] Let *m*_*0*_ denote the entire-sample prediction model and *s*_*0*_ denote all visits in the development dataset. Within each bootstrap sample, ${s}^{(b)}$, a random forest prediction model for suicide, ${m}^{(b)}$, was estimated using the tuning parameters selected by cross-validation for the entire-sample model. Then, the optimism of each within-sample performance measure was calculated as follows, using AUC as an example:

$${Optimism}_{AUC}^{(b)}=AUC\left({m}^{\left(b\right)}, {s}^{\left(b\right)}\right)-AUC({m}^{\left(b\right)}, {s}_{0})$$

where $AUC(m, s)$ is the AUC of model $m$ evaluated in sample $s$. For measures of classification accuracy, thresholds were defined at specified percentiles of the distribution of in-sample risk predictions for ${s}_{0}$ in the entire-sample model when evaluating performance of ${m}_{0}$ and with respect to the distribution of in-sample risk predictions for ${s}^{(b)}$ when evaluating the performance of the prediction model ${m}^{(b)}$.

A total of $B=500$ event-stratified person-level bootstrap samples were drawn from the development dataset. Optimism-corrected estimates were then calculated for each performance metric. For example, the optimism-corrected, ${AUC}_{corrected},$ was calculated as follows:

$${AUC}_{corrected}=AUC\left({m}_{o}, {s}_{0}\right)- \frac{1}{B}\sum_{b=1}^{B}{Optimism}_{AUC}^{(b)}$$

Quantile-based 95% CIs were estimated from the distribution of optimism-corrected performance measures across bootstrap samples [30].

### Prospective validation

Performance of prediction models estimated in the split-sample training set and in the entire-sample development dataset was evaluated in the prospective validation dataset. Quantile-based 95% CIs were estimated from 1,000 person-level event-stratified bootstrap samples. Estimated prospective performance of the split-sample and entire-sample prediction models were compared to identify which model best predicted suicide in a prospective sample. Internal validation estimates for the split-sample model and for the entire-sample model obtained by cross-validation and bootstrap-optimism correction were compared to estimated performance of each model in the prospective validation set to evaluate whether each method adequately adjusted for overfitting.

Further description of study methods can be found in the Additional Analytic Details in the Supplemental Materials.

## Results

### Data description

Our analysis included 9,610,318 visits in the development dataset (2009–2014) and 3,754,137 visits in the prospective validation dataset (2015–2017). Suicides were observed following 2,318 visits in the development data (a rate of 24 events per 100,000 visits) and following 710 visits in the prospective validation data (19 events per 100,000 visits) (Table S4).

Characteristics of patients at the time of visit are presented in Table 1. The majority of visits in the development set were for patients who were female (64.1%), White (69.0%), non-Hispanic (77.3%), had commercial insurance (75.7%), and had a depression (75.5%) or anxiety (70.1%) diagnosis or antidepressant prescription (67.9%) in the 5 years preceding the visit. Some changes in characteristics were observed over time, as is anticipated in the course of routine clinical care. Visits in the prospective sample were more likely to be made by patients with Medicaid insurance (11.0% vs. 4.2% in development data) and were more likely to have a PHQ-9 recorded in the past year (43.3% vs. 13.5% in development data). Increases in PHQ-9 utilization and the proportion of respondents reporting no suicidal ideation (6.1% in the development data vs. 19.8% in the prospective validation) were expected because PHQ-9 use was by discretion in the earlier study years (and providers typically used it with patients with more severe symptoms) and, in later years, routine administration for all visits was recommended by some data-contributing health systems.

**Table 1:** Characteristics of mental health specialty visits for development sample (January 1, 2009 – September 30, 2014) and prospective validation sample (January 1, 2015—September 30, 2017)

Characteristic	Development dataset (*N* = 9,610,318 visits)		Prospective validation dataset (*N* = 3,754,137 visits)	
	N	%	N	%
Female	6,081,526	64.1	2,406,695	64.1
Age				
17 or younger	1,048,533	10.9	417,977	11.1
18–29	1,562,616	16.3	670,337	17.9
30–44	2,453,898	25.5	948,716	25.3
45–64	3,510,438	36.5	1,242,067	33.1
65 or older	1,034,813	10.8	475,040	12.7
Race				
White	6,627,544	69.0	2,511,547	66.9
Asian	448,590	4.7	192,284	5.1
Black	836,479	8.7	324,852	8.7
Hawaiian/Pacific Islander	108,410	1.1	32,803	0.9
Native American	92,312	1.0	35,824	1.0
More than one	3,298	0.03	1,788	0.1
Other race recorded	48,800	0.5	20,879	0.6
Not race recorded	1,444,885	15.0	634,160	16.7
Ethnicity				
Hispanic	2,183,742	22.7	987,061	26.3
Insurance type				
Commercial group	7,275,199	75.7	2,544,926	67.8
Individual	325,139	3.4	141,234	3.8
Medicare	1,409,742	14.7	572,476	15.2
Medicaid	406,606	4.2	413,007	11.0
Other	193,632	2.0	82,494	2.2
PHQ-9 9^th^ item response^1^				
0, Not at all	590,468	6.1	743,288	19.8
1, Several days	121,483	1.3	153,671	4.1
2, More than half the days	43,697	0.5	46,977	1.3
3, Nearly every day	31,408	0.3	32,410	0.9
Not recorded	8,823,262	91.8	2,777,791	74.0
PHQ-9 recorded in the previous year	1,296,856	13.5	1,623,943	43.3
Diagnoses in past 5 years, including index visit				
Depression	7,257,525	75.5	2,852,694	76.0
Anxiety	6,734,366	70.1	2,967,524	79.0
Bipolar depression	1,317,629	13.7	474,897	12.6
Schizophrenia	400,713	4.2	154,590	4.1
Other psychosis	537,050	5.6	221,351	5.9
Dementia	96,751	1.0	54,782	1.5
Attention deficit disorder	1,151,179	12.0	485,877	12.9
Autism spectrum disorder	131,900	1.4	65,795	1.8
Personality disorder	1,961,027	20.4	603,400	16.1
Alcohol use disorder	1,561,302	16.2	553,027	14.7
Drug use	1,654,129	17.2	588,481	15.7
Post-traumatic stress disorder	845,842	8.8	400,959	10.7
Eating disorder	350,320	3.6	144,204	3.8
Traumatic brain injury	312,516	3.3	140,940	3.8
Prescription fills in past 5 years				
Antidepressants	6,521,196	67.9	2,559,147	68.2
Benzodiazepines	4,536,960	47.2	1,641,900	43.7
Hypnotics	1,427,849	14.9	381,353	10.2
2nd generation antipsychotics	2,035,084	21.2	799,401	21.3
Encounters in prior 5 years with mental health diagnosis				
Inpatient	2,296,579	23.9	859,640	22.9
Outpatient	8,789,642	91.5	3,431,776	91.4
Emergency department	3,167,119	33.0	1,347,840	35.9
Suicide attempt in prior 5 years	381,591	4.0	172,329	4.6
Charlson comorbidity index				
0	7,133,017	74.2	2,719,729	72.4
1	1,402,435	14.6	565,038	15.1
> 1	1,074,866	11.2	469,370	12.5
Suicide death within 90 days of visit	2318	24 per 100,000	710	19 per 100,000

^1^The PHQ-9 9^th^ item asks patients about the frequency of thoughts of suicide or death in the prior 2 weeks

### Comparison of methods for prediction model estimation and internal validation

As a reminder, we have two classes of approaches: The split-sample approach only uses a subset of the development dataset to construct our prediction model and evaluates performance on the remaining subset; the entire-sample approach constructs our prediction model on the entirety of our development dataset and uses fivefold cross-validation or the bootstrap to evaluate performance. These two classes of approaches differ both in the prediction model they estimate and how they evaluate performance. This is important in interpreting the results, as we are interested in selecting a strategy that both a) results in a strong prediction model, as demonstrated with good performance on the prospective validation set, as well as b) gives an accurate internal estimate of the prospective validation performance. We first report performance of the split-sample and entire-sample suicide prediction models in the prospective dataset; we then present optimism of internal validation estimates compared to prospective performance.

Prediction models estimated with a subset of the development data and in the entire development dataset showed similar discrimination in the prospective validation dataset. The split-sample model had an AUC of 0.814 (95% CI: 0.771–0.851) in the prospective validation set, and the entire-sample model had an AUC of 0.811 (0.768–0.849) (Table 2). Sensitivity (Table 3) and PPV (Table 4) of the split-sample and entire-sample models were also similar in the prospective validation set. For example, at the 90^th^ percentile, prospective sensitivity was 52.0% (41.7–61.1%) for the split-sample prediction model and 53.0% (43.0–62.3%) for the entire-sample prediction model. At the same threshold, PPV was 7.9 events per 100,000 visits (5.8–10.5 events per 100,000 visits) for the split-sample model and 9.1 events per 100,000 visits (6.7–12.0 events per 100,000 visits) for the entire-sample model. Prospective performance of the two models was also similar for specificity (Table S5).

**Table 2:** Estimated AUC of prediction models from split-sample and entire sample estimation approaches in the development dataset and prospective validation dataset

**Prediction model estimation**	**Internal validation approach**	**AUC** (95% CI)	
		**Development dataset**	**Prospective dataset**
**Split-sample**	Validate in testing set	0.846 (0.817, 0.870)	0.814 (0.771, 0.851)
**Entire-sample**	Cross-validation	0.832 (0.812, 0.851)	0.811 (0.768, 0.849)
	Bootstrap optimism correction	0.878 (0.861, 0.890)

**Table 3:** Sensitivity (95% CI) of prediction models from split-sample and entire sample estimation approaches in the development dataset and prospective validation dataset

	Split-sample prediction model		Entire-sample prediction model		
Risk percentile cutpoint	Testing set, Development	Prospective validation	5-fold cross-validation, Development	Bootstrap optimism correction, Development	Prospective validation
≥ 99%	12.2% (7.5%, 17.8%)	19.9% (10.9%, 30.1%)	11.6% (8.4%, 15.2%)	17.5% (8.8%, 24.6%)	15.1% (7.5%, 24.2%)
≥ 95%	42.1% (32.6%, 51.0%)	40.1% (30.1%, 50.2%)	33.9% (27.1%, 40.5%)	47.2% (40.1%, 52.9%)	39.2% (28.7%, 50.5%)
≥ 90%	54.0% (45.4%, 62.1%)	52.0% (41.7%, 61.1%)	50.5% (43.3%, 57.2%)	63.2% (56.3%, 67.8%)	53.0% (43.0%, 62.3%)
≥ 75%	78.5% (72.9%, 83.4%)	75.1% (67.0%, 82.3%)	75.1% (70.4%, 79.8%)	84.4% (81.0%, 86.9%)	72.1% (63.8%, 79.7%)

**Table 4:** Positive predictive value per 100,000 visits (95% CI) of prediction models from split-sample and entire sample estimation approaches in the development dataset and prospective validation dataset

	Split-sample prediction model		Entire-sample prediction model		
Risk percentile cutpoint	Testing set, Development	Prospective validation	5-fold cross-validation, Development	Bootstrap optimism correction, Development	Prospective validation
≥ 99%	27.5 (16.7, 39.3)	27.6 (14.3, 46.3)	30.0 (21.9, 39.3)	41.7 (21.0, 58.9)	23.7 (11.4, 40.7)
≥ 95%	19.3 (13.3, 26.5)	12.6 (8.6, 17.5)	16.0 (12.4, 19.7)	22.6 (19.8, 25.2)	13.9 (9.4, 19.9)
≥ 90%	12.2 (9.0, 15.9)	7.9 (5.8, 10.5)	12.0 (9.9, 14.4)	15.2 (13.8, 16.3)	9.1 (6.7, 12.0)
≥ 75%	7.1 (5.8, 8.7)	4.8 (3.9, 5.9)	7.2 (6.3, 8.2)	8.1 (7.8, 8.4)	4.9 (3.9, 6.0)

Internal validation estimates obtained in the split-sample testing set and cross-validation in the entire-sample better reflected prospective performance than using bootstrap optimism-correction. The cross-validation estimate of AUC in the entire-sample model (0.832, 95% CI:0.812, 0.851) was closest to the prospective AUC (Table 2), indicating that this internal validation approach best estimated future model performance. The spit-sample testing estimate of AUC (0.846, 95% CI: 0.817–0.870) was farther from the prospective AUC of the split-sample model, but the 95% CIs for each overlapped. In contrast, the bootstrap optimism-corrected estimate of AUC in the entire-sample model (0.878, 95% CI: 0.861–0.890) over-estimated prospective discrimination.

Interval validation estimates of sensitivity (Table 3) and specificity (Table S5) also show that split-sample testing and entire-sample cross-validation better estimate prospective performance than bootstrap optimism correction. For example, at the 90^th^ percentile, split-sample testing and entire-sample cross-validation estimates of sensitivity (54.0% and 50.5%, respectively) are similar to the prospective sensitivities (52.0% and 53.0%, respectively) while the bootstrap optimism correction estimate (63.2%) over-estimated sensitivity of the entire-sample model.

Because the overall event rate was lower in the prospective validation set, all internal validation methods overestimated PPV. As seen in other performance metrics, overestimation was greatest for the bootstrap optimism corrected estimate of PPV for the entire-sample model and more modest for the split-sample testing and entire-sample cross-validation estimates of PPV (Table 4).

## Discussion

In this case study, we empirically evaluated approaches for prediction model estimation and internal validation in the context of predicting risk of suicide following an outpatient mental health visit. Our first aim was to compare performance of a prediction model estimated with random forests in a 50% randomly sampled training subset of a development dataset containing all visits from January 20,019- September 2014 from seven health systems (split-sample prediction model) to that of a random forest model estimated in the entire development dataset (entire-sample model). We found that performance of the entire-sample and split-sample prediction models were comparable in the prospective validation dataset containing all visits from 2015–2017; using all visits in the development dataset for estimation conferred no advantage with respect to risk discrimination or classification accuracy. In these analyses, the different performance metrics examined agreed on the relative performance of the models being compared. In practice, this is not always the case, thus we recommend study teams select a primary performance metric that reflects both how the prediction model will be used in a clinical setting as well as the priorities of stakeholders before conducting analyses so that conclusions may focus on that measure of performance.

Our second aim was to evaluate whether three validation approaches adequately adjusted for overfitting. Internal estimates of prediction model performance were compared to prospective performance to assess optimism. While we expect performance in future data may degrade somewhat due to non-random differences in patterns of care (like those seen between the development and prospective development samples in Table 1), the internal validation approach that minimizes optimism is preferable. Overestimation was greatest for optimism-corrected estimates of performance for the entire-sample model obtained via the bootstrap. Optimism was meaningfully smaller for cross-validated estimates of entire-sample prediction model performance and split-sample model validation in the testing set.

While both approaches are preferable to bootstrap optimism correction, we identified two benefits of cross-validation for the entire-sample model over testing set validation for the split-sample model. First, using the entire sample to assess performance via cross-validation results in more narrow confidence intervals than evaluating performance in only a subset of observations. For example, the 95% CI for the split-sample model AUC evaluated in the testing set was 36% wider than the cross-validated 95% CI for the AUC of the entire-sample model (Table 2). Second, the AUC estimated in the testing set of the split-sample model was greater (and, accordingly, more optimistic) than the cross-validated AUC for the entire-sample model. A disadvantage of split-sample prediction model estimation and validation is that, because data are divided into training and testing sets only once, estimates of performance elicited from the testing set may be more sensitive to random variation in the data splitting.

In this study, optimism-corrected performance estimates obtained via bootstrap did not adequately adjust for overfitting when evaluating performance of a prediction model estimated with the entire development dataset. When developing a prediction model to be used in clinical care, it is imperative to have accurate internal validation of predictive performance to guide decisions about whether, and how to, implement it. Moreover, relying on bootstrap optimism correction for internal validation would lead researchers to incorrectly conclude that the prediction model estimated in the entire sample better predicted suicide risk than the model estimated and validated with a split-sample approach. Researchers typically evaluate several possible prediction models and select the model with the best validation performance. Correct internal estimates of prediction model performance are needed to inform this decision.

Our findings stand in contrast with prior research demonstrating the validity of bootstrap optimism correction for internal validation of logistic regression prediction models estimated with a relatively small sample size and number of predictors. Characteristics of this study vary from that context in several ways: we used several million observations and over one hundred predictors to estimate random forest models to predict a rare event. Our study was not designed to identify which of these elements (modeling method, event rate, sample size, or number of predictors) is the reason that bootstrap optimism correction failed in this setting. We expect that bootstrap optimism correction did not adequately quantify prediction model optimism because each bootstrap sample contains, on average, 63.2% of the observations in the original sample. So, comparing the in-sample performance of a prediction model estimated with the bootstrap sample to its performance in the original sample does not provide an out-of-sample assessment. Other bootstrap-based optimism correction approaches, including the 0.632 method [15] and the 0.632 + method, [13] also incorporate in-sample observations when estimating performance and, as such, may overestimate performance in this setting. By comparison, cross-validation and using an independent testing set for validation each ensure no observations are used for both model estimation and validation.

Data-driven machine learning methods designed to balance bias and variance can fit very complex models (incorporating interactions and non-linear relations). As datasets grow in size, these methodologies learn increasingly complex models. In contrast, the complexity of simple parametric models, like linear logistic regression does not change (as there is no bias/variance tradeoff). The example explored here suggests that this difference is key: while bootstrap optimism correction is adequate for simpler non-adaptive models, internal validation with out-of-sample observations (either a split-sample approach or cross-validation on the entire sample) may be needed for more complex models that are trained to balance bias and variance. While considering only one example dataset and a single machine learning method are limitations of our study, the undesirable properties of bootstrap optimism correction in this case study point to limitations of the method not previously explored in the clinical prediction modeling literature. There is an opportunity for future study of internal validation methods for other non-parametric modeling approaches, such as boosting and artificial neural networks.

## Conclusions

This case study illustrates an example where bootstrap optimism correction did not provide accurate internal validation. While we cannot conclude this method is inaccurate for a broad class of problems (e.g., any non-parametric model), we suggest caution if using bootstrap optimism correction in a similar setting and recommend comparing optimism-corrected performance estimates to those obtained from an out-of-sample method, such as cross-validation.

## Supplementary Information

**Additional file 1:** **Table S1.** Data availability dates for participating sites. **Table S2. **List of all predictors for random forest models. **Table S3.** Cross-validated AUC estimates for tuning parameter selection for (a) split-sample prediction model and (b) entire-sample prediction model. **Table S4.** Description of development and prospective sample. **Table S5.** Specificity (95% CI) of prediction models from split-sample and entire sample estimation approaches in the development dataset and prospective validation dataset. **Figure S1.** Moving estimate of (a)cross-validated and (b) bootstrap optimism-corrected AUC (95% CI), 1-500 bootstrap samples. **Figure S2.** Moving estimate of (a) cross-validated and (b) bootstrap optimism-corrected sensitivity (95% CI) above 99th percentile threshold, 1-500 bootstrap samples.

## Data Availability

The datasets generated and analyzed during this study are not publicly available because they contain detailed information from the electronic health records in the health systems participating in this study and are governed by Health Insurance Portability and Accountability Act (HIPAA). Data are, however, available upon reasonable request with permission of all health systems involved and a fully executed data use agreement. Contact corresponding author (Yates Coley, rebecca.y.coley@kp.org) with data inquiries.

## Abbreviations

AUC: Area under the curve
CI: Confidence interval
NPV: Negative predictive value
PHQ-9: Patient Health Questionnaire 9-item
PPV: Positive predictive value
