# Supplementary material for: Empirical evaluation of internal validation methods for prediction in large-scale clinical data with rare-event outcomes: a case study in suicide risk prediction
Source: BMC Med Res Methodol. 2023 Feb 1;23:33. doi: 10.1186/s12874-023-01844-5 (PMC9890785; doi:10.1186/s12874-023-01844-5)
Supplement: Supplementary file 1 — Additional file 1: Table S1. Data availability dates for participating sites. Table S2. List of all predictors for random forest models. Table S3. Cross-validated AUC estimates for tuning parameter selection for (a) split-sample prediction model and (b) entire-sample prediction model. Table S4. Description of development and prospective sample. Table S5. Specificity (95% CI) of prediction models from split-sample and entire sample estimation approaches in the development dataset and prospective validation dataset. Figure S1. Moving estimate of (a)cross-validated and (b) bootstrap optimism-corrected AUC (95% CI), 1-500 bootstrap samples. Figure S2. Moving estimate of (a) cross-validated and (b) bootstrap optimism-corrected sensitivity (95% CI) above 99th percentile threshold, 1-500 bootstrap samples. [file 12874_2023_1844_MOESM1_ESM.docx]

Supplemental Materials for “Empirical evaluation of internal validation methods for prediction in large-scale clinical data with rare-event outcomes: a case study in suicide risk prediction”

p. 2 Additional analytic details

p. 7 **Table S1:** Data availability dates for participating sites

p. 8 **Table S2:** List of all predictors for random forest models

p. 14 **Table S3:** Cross-validated AUC estimates for tuning parameter selection for (a) split-sample prediction model and (b) entire-sample prediction model

p. 15 **Table S4:** Description of development and prospective sample

p. 16 **Table S5:** Specificity (95% CI) of prediction models from split-sample and entire sample estimation approaches in the development dataset and prospective validation dataset

p. 17 **Figure S1:** Moving estimate of (a) cross-validated and (b) bootstrap optimism-corrected AUC (95% CI), 1-500 bootstrap samples

p. 19 **Figure S2:** Moving estimate of (a) cross-validated and (b) bootstrap optimism-corrected sensitivity (95% CI) above 99^th^ percentile threshold, 1-500 bootstrap samples

p. 21 References

**Additional analytic details** on our analytic approach for estimating and validating suicide prediction models.

Study setting: Seven health systems contributed data for this analysis: HealthPartners; Henry Ford Health System; and the Colorado, Hawaii, Northwest, Southern California, and Washington regions of Kaiser Permanente. Each site is a member of the Mental Health Research Network and, as such, maintains a research data warehouse following the Health Care Systems Research Network’s Virtual Data Warehouse (VDW) model.^1^ The VDW contains insurance enrollment records, electronic health records, insurance claims, dispensed prescriptions, state mortality records, and census-derived neighborhood characteristics.

Dataset, inclusion and exclusion criteria: Analysis includes all outpatient mental health visits at the participating health systems for patients age 13 years or older between January 1, 2009 and September 30, 2017 with complete capture of cause of death data. (Dates for availability of cause of death data are in Table S1.) Patients did not need to be enrolled in the health system at the time of the visit, nor did they need to remain enrolled for the 90-day outcome follow-up window because health systems have access to mortality records for all patients. Because many patients had multiple outpatient mental health visits during the study period, the analytic dataset may contain more than one visit per patient.

The sample was divided into a development dataset containing all visits from January 1, 2009-September 30, 2014 and a prospective validation dataset containing visits from January 1, 2015- September 30, 2017. The development dataset was used for prediction model estimation and internal validation. The prospective validation dataset was used to evaluate future performance of prediction models estimated in the development dataset. Visits from October 1, 2014- December 31, 2014 were discarded to ensure no suicides appeared in both datasets.

*Rationale for visit-level sampling framework:* We estimated prediction models for suicide risk following an outpatient mental health visit (rather than predicting risk on the person level) because risk of suicide varies over time within a person. Our data span several years in which people may have multiple suicide attempts, as well as periods of time with lower suicide risk. The goal of developing suicide risk prediction models is to ultimately use them in a clinical setting to guide suicide prevention interventions at the time of a visit. Suicide prevention interventions have both a time and financial cost, so they are not provided to a person indefinitely, and they do not alter risk permanently. So, determining when to give an intervention is as important as determining who should receive it.

Outcome: Suicide deaths in the 90 days following an outpatient mental health visit were identified from state death certificates indicating definite or probable suicides. In the case of a patient having multiple visits within 90 days, the outcome follow-up windows for those visits overlap. The same suicide death may be preceded by more than one visit in the 90 days beforehand. As a result, the number of visits with a suicide death within 90 days will be greater than the number of unique suicide deaths observed.

Visit dates for the development and prospective validation datasets were defined with a 90-day gap so that there are no suicide deaths appearing in both the development and prospective samples.

*Rationale for including injuries or poisonings with undetermined intent:* Including these deaths in the outcome definition increases ascertainment of probable suicide deaths by 5-10% in Mental Health Research Network sites.^2, 3^ This outcome definition has also been evaluated outside of participating health systems.^4, 5^

*Rationale for 90-day outcome window:* The 90-day time window for observing suicide deaths was defined in collaboration with clinical leaders. While using a 30-day follow-up period reduces the overlap in follow-up across visits (and resulting correlated outcomes), it does not remove this statistical concern. Using a shorter time window also reduces the event rate substantially, which reduces statistical power to estimate models that discriminate risk well and reduces the precision of model validation estimates. (Here, statistical power refers to the likelihood that true predictors of the outcome will be identified, avoiding type II errors, and risk will be estimated accurately.) The 90-day follow-up window was also preferred by our clinical collaborators because many of the empirically supported interventions to address suicide risk (e.g., Dialectical Behavior Therapy, safety planning) would be expected to take effect over that period.

*Rationale for a binary vs. time-to-event outcome:* We chose to model suicide death as a binary outcome rather than a time-to-event outcome for several reasons. First, censored follow-up was not a concern because all visits in the analysis had mortality data available for 90 days following the visit. Second, there are many more computationally efficient options for estimating prediction models for large datasets—our data included several million visits and over 100 predictors--with binary outcomes than with survival outcomes. Third, while modeling suicide as a time-to-event outcome following a visit would allow for censoring follow-up at the time of the next visit (and, thus, avoid overlapping follow-up windows), censoring would be informative (people with more mental health visits have higher suicide risk) and violate the independent censoring assumption required of standard survival models; we are not aware of options for estimating partially conditional survival models (as are needed to accommodate informative censoring) with big data. With a binary outcome, we were able to examine a prediction model estimation and validation with a tree-based ensemble model (random forest).

Predictors: Predictor definitions and interactions were specified in advance of conducting the analysis and followed those used in Simon et al (2018).^3^ All predictors are reported in Table S2.

Missing and incomplete data: Outcome status was not missing for any visit, as each site’s VDW contains mortality data from state and national death registries. Missing predictor information was captured using indicator variables, e.g., a binary indicator that there was no response to the 9^th^ item on the PHQ-9.

Rates of PHQ-9 response^6^ changed during the study period. Earlier in the study period, use of the PHQ-9 was at provider discretion; later, most health systems contributing data to this sample recommended use of the PHQ-9 for all outpatient mental health visits. PHQ-9 surveys with a minimum of 6 items completed were included in a patient’s clinical history while those with fewer than 6 items completed were discarded. When response to one or more of the first eight items (PHQ-8) is missing, the total PHQ-8 score is taken to be the average response to all completed items (excluding the 9^th^ item) multiplied by eight. The 9^th^ item, which asks specifically about suicidal ideation, is summarized separately and includes a category for missing responses.

Observation of clinical history was incomplete for people without 5 years of prior health insurance enrollment prior to the study visit, so recorded prior diagnoses, prescriptions, encounters, and comorbidities were under-counted. Our analysis included all visits from patients with shorter enrollment because we wanted to estimate prediction models that could be used to guide care for all patients (not just those with 5 years of enrollment) and because lack of continuous, long-term health insurance enrollment is associated with other social determinants of health (e.g., stable employment) that may be risk factors for suicide. Duration of health plan coverage at the time of the study visit was also included as a predictor. Work conducted by our research group after the starting the project presented here has found that more complex variable definitions (e.g., counting the number of times a diagnosis was present in a patient’s history vs. using a binary indicator of any diagnosis) and variable definitions that adjusted for length of enrollment (e.g., the proportion of months enrolled in which a diagnosis was present) did not improve performance of suicide prediction models.

Prediction model estimation with random forest: We used random forest models to estimate the probability of 90-day suicide.^7^ Random forests apply a bootstrap aggregating technique: bootstrap resampling is done to obtain samples in which to estimate each classification tree in the random forest. We stratified this resampling on outcome status to maintain a constant event rate across trees. The Gini index, which measures node impurity, was used as the node-splitting rule.^8^ Each tree returned estimated probabilities equal to the proportion of events among visits in each terminal node. Predicted suicide risk for a visit was obtained by averaging predictions from each tree in the random forest.

With each sample used for model estimation, 5-fold cross-validation was used to select the tuning parameter combination that optimized out-of-fold area under the curve (AUC).^9^ People, rather than visits, were randomly assigned to folds (so that all visits from a person appeared in the same fold).^10^ Cross-validation folds were additionally stratified by event status, to obtain an equal number of people with suicide across the folds. We estimated random forest models with 100 trees because our prior analyses with these data have shown improvements in performance plateau after about 50 trees.

We used the ranger package in R to estimate random forest models.^8, 11^

Tuning parameter selection: The split-sample prediction model was estimated in the training dataset using a random forest model with tuning parameters selected by cross-validation: 5 predictors randomly sampled at each node and a minimum terminal node size of 25,000. The out-of-fold AUC obtained by cross-validation was 0.811 (Table S3(a)). The entire-sample random forest prediction model optimal tuning parameters were 11 predictors sampled at each node and a minimum terminal node size of 100,000 (out-of-fold AUC=0.832, Table S3(b)).

Performance measures: We evaluated predictive accuracy using several common measures—area under the curve, sensitivity, specificity, and positive predictive value (PPV). AUC quantifies risk discrimination and provides a convenient summary of how well risk is ordered across the entire sample. As clinical implementation of suicide prediction models in health systems generally use a binary risk prediction (patient currently at higher risk or suicide vs. not), we also examined measures of classification accuracy (sensitivity, specificity, and PPV) at the 99^th^, 95^th^, 90^th^, and 75^th^ percentiles of the distribution of predictions in the training dataset.

Due to space constraints, specificity results are included in the supplement (Table S5), rather than in the primary manuscript. Specificity in the prospective validation set was similar for the split-sample and entire-sample prediction models. Estimates of specificity in the development dataset were similar for all internal validation approaches.

We did not evaluate negative predictive value (NPV) because, since suicide is a rare event, the NPV is nearly one for a binary prediction rule defined at all risk thresholds examined.

NB on PPV: PPV can be an informative measure to consider when balancing benefits and harms of a treatment in a population, but there is disagreement over what PPV is adequate for suicide prediction models.^12, 13^ PPV is also influenced by the event rate in a population, with a higher event rate corresponding to a greater PPV. Because the event rate was lower in our prospective validation set, PPV was overestimated in the development dataset by all internal validation methods, that is, our comparison of estimated PPV between the development and prospective validation datasets quantifies this temporal trend as well as the optimism of internal validation estimates. Despite this limitation, we included PPV estimates because this measure of performance is clinically relevant.

The Brier score, equivalent to the mean squared error for a binary outcome is often used as an alternative to the AUC in rare event prediction because the AUC can be sensitive to small changes in prediction when there are a small number of events observed. Similar to PPV, however, the Brier score is also influenced by event rate, such that the Brier score evaluated in the prospective validation set would also reflect the rate of suicide death. Given this limitation and that the Brier score is not commonly used by clinical stakeholders when deciding whether or not to implement a prediction model, we did not examine Brier score for this analysis.

Finally, we declined to examine prediction model calibration because it is not relevant to how models for suicide risk prediction models are used in practice. Continuous risk scores are used to select thresholds for intervention based on health system capacity and expected benefit for the planned intervention, but absolute estimated risks are not used to inform care. Moreover, calibration is a secondary measure of model performance relative to discrimination and classification accuracy because prediction models can easily be re-calibrated so that predictions better align with observed rates.

Alternate measures of optimism: In *Clinical prediction models: A practical approach to development, validation, and updating*, Steyerberg also proposes an “approximate optimism correction” method that uses a similar bootstrapping approach to the “exact” bootstrap optimism correction method examined in this manuscript.^14^ This approximate approach takes the average across all bootstrap samples $b=1,\ldots, B$of the performance a prediction model estimated in the bootstrap sample, $m^{(b)},$ as evaluated in the original development dataset *s_0_*. For example, the approximate optimism-corrected AUC is:

$${AUC}_{approximate}=\frac{1}{B}\sum_{b=1}^{B} AUC(m^{\left( b \right)}, s_{0}).$$

Initial analyses with this approach produced estimates similar to those obtain via bootstrap optimism correction presented in this paper, that is, approximate optimism-corrected estimates of entire-sample model performance overestimated prospective performance.

Stability of optimism estimated via bootstrap: Bootstrap resampling was repeated 500 times for both entire-sample validation methods to reduce computational burden. Moving estimates of the mean and 95% CI were monitored to ensure estimate stability after 500 bootstrap samples. Figures S1 and S2 show moving estimates for AUC and sensitivity above the 99^th^ risk percentile threshold.

**Table S1:** Data availability dates for participating sites

| **Health system** | **Data start date** | **Last date with complete cause of death data**^a^ |
| --- | --- | --- |
| HealthPartners | January 1, 2009 | December 31, 2016 |
| Henry Ford Health System | December 1, 2012^b^ | December 31, 2015 |
| Kaiser Permanente Colorado | January 1, 2009 | December 31, 2017 |
| Kaiser Permanente Hawaii | January 1, 2009 | December 31, 2016 |
| Kaiser Permanente Northwest | January 1, 2009 | December 31, 2016 |
| Kaiser Permanente Southern California | January 1, 2009 | December 31, 2016 |
| Kaiser Permanente Washington | January 1, 2009 | December 31, 2016 |

^a^ The study sample includes visits up to September 30 of the year with complete capture of cause of death data to allow for 90 days follow-up after outpatient mental health visits. For example, visits through September 30, 2016 are included for health systems with cause of death data complete through December 31, 2016.

^b^ Only visits that occurred after the implementation of a new electronic health records system at Henry Ford were included in the sample

**Table S2:** List of all predictors for random forest models

| **Variable Name** | **Details** |
| --- | --- |
| Age | Age in years at index visit |
| Female | Gender=Female |
| dep_dx_pre5y_noi_cumulative | Depression diagnosis (DX), (0,5] years prior to index visit |
| dep_dx_pre5y | Depression DX, [1,5] years prior |
| anx_dx_pre5y_noi_cumulative | Anxiety DX, (0,5] years prior |
| anx_dx_pre5y | Anxiety DX, [1,5] years prior |
| bip_dx_pre5y_noi_cumulative | Bipolar depression DX, (0,5] years prior |
| bip_dx_pre5y | Bipolar depression DX, [1,5] years prior |
| sch_dx_pre5y_noi_cumulative | Schizophrenia DX, (0,5] years prior |
| sch_dx_pre5y | Schizophrenia DX, [1,5] years prior |
| oth_dx_pre5y_noi_cumulative | Other Psychosis DX, (0,5] years prior |
| oth_dx_pre5y | Other Psychosis DX, [1,5] years prior |
| dem_dx_pre5y_noi_cumulative | Dementia DX, (0,5] years prior |
| dem_dx_pre5y | Dementia DX, [1,5] years prior |
| add_dx_pre5y_noi_cumulative | Attention Deficit Disorder (ADD) DX, (0,5] years prior |
| add_dx_pre5y | ADD DX, [1,5] years prior |
| asd_dx_pre5y_noi_cumulative | ASD DX, (0,5] years prior |
| asd_dx_pre5y | ADD DX, [1,5] years prior |
| per_dx_pre5y_noi_cumulative | Personality disorder DX, (0,5] years prior |
| per_dx_pre5y | Personality disorder DX, [1,5] years prior |
| alc_dx_pre5y_noi_cumulative | Alcohol use disorder DX, (0,5] years prior |
| alc_dx_pre5y | Alcohol use disorder DX, [1,5] years prior |
| pts_dx_pre5y_noi_cumulative | Post-traumatic stress disorder (PTSD) DX, (0,5] years prior |
| pts_dx_pre5y | PTSD DX, [1,5] years prior |
| eat_dx_pre5y_noi_cumulative | Eating disorder DX, (0,5] years prior |
| eat_dx_pre5y | Eating disorder DX, [1,5] years prior |
| tbi_dx_pre5y_noi_cumulative | Traumatic brain injury (TBI) DX, (0,5] years prior |
| tbi_dx_pre5y | TBI DX, [1,5] years prior |
| dru_dx_pre5y_noi_cumulative | Drug use disorder DX, (0,5] years prior |
| dru_dx_pre5y | Drug use disorder DX, [1,5] years prior |
| antidep_rx_pre3m | Antidepressant prescription (rx) (0,3] months |
| antidep_rx_pre1y_cumulative | Antidepressant rx (0, 1] year |
| antidep_rx_pre5y_cumulative | Antidepressant rx (0, 5] years |
| benzo_rx_pre3m | Benzodiazepine rx (0,3] months |
| benzo_rx_pre1y_cumulative | Benzodiazepine rx (0,1] year |
| benzo_rx_pre5y_cumulative | Benzodiazepine rx (0,5] years |
| hypno_rx_pre3m | Hypnotic rx (0,3] months |
| hypno_rx_pre1y_cumulative | Hypnotic rx (0,1] year |
| hypno_rx_pre5y_cumulative | Hypnotic rx (0,5] years |
| sga_rx_pre3m | Second Generation Antipsychotic (SGA) rx (0,3] months |
| sga_rx_pre1y_cumulative | SGA rx (0,1] year |
| sga_rx_pre5y_cumulative | SGA rx (0,5] years |
| mh_ip_pre3m | Inpatient hospitalization with mental health diagnosis (IP with MH dx) (0, 3] months |
| mh_ip_pre1y_cumulative | IP with MH dx (0, 1] year |
| mh_ip_pre5y_cumulative | IP with MH dx (0, 5] years |
| mh_op_pre3m | IP with MH dx (0, 3] months |
| mh_op_pre1y_cumulative | IP with MH dx (0, 1] year |
| mh_op_pre5y_cumulative | IP with MH dx (0, 5] years |
| mh_ed_pre3m | Emergency Department (ED) utilization with MH dx (0, 3] months |
| mh_ed_pre1y_cumulative | ED with MH dx (0, 1] year |
| mh_ed_pre5y_cumulative | ED with MH dx (0, 5] years |
| any_sui_att_pre3m | Prior suicide attempt (0, 3] months |
| any_sui_att_pre1y_cumulative | Prior suicide attempt (0, 1] year |
| any_sui_att_pre5y_cumulative | Prior suicide attempt (0, 5] years |
| lvi_sui_att_pre3m | Laceration-based violent (LVI) suicide attempt (0, 3] months |
| lvi_sui_att_pre1y_cumulative | LVI suicide attempt (0, 1] year |
| lvi_sui_att_pre5y_cumulative | LVI suicide attempt (0, 5] years |
| ovi_sui_att_pre3m | Other (not laceration-based) violent (OVI) suicide attempt (0, 3] months |
| ovi_sui_att_pre1y_cumulative | OVI suicide attempt (0, 1] year |
| ovi_sui_att_pre5y_cumulative | OVI suicide attempt (0, 5] years |
| any_inj_poi_pre3m | Any prior injury or poisoning (0, 3] months |
| any_inj_poi_pre1y_cumulative | Any prior injury or poisoning (0, 1] year |
| any_inj_poi_pre5y_cumulative | Any prior injury or poisoning (0, 5] years |
| any_sui_att_pre5y_cumulative_f | Prior suicide attempt (0, 5] years * (gender=F) |
| any_sui_att_pre5y_cumulative_a | Prior suicide attempt (0, 5] years * (age) |
| charlson_score | Charlson comorbidity score on index date |
| charlson_a | Charlson score on index date * (age) |
| charlson_mi | Charlson indicator myocardial infaction |
| charlson_chd | Charlson indicator coronary heart disease |
| charlson_pvd | Charlson indicator peripheral vascular disease |
| charlson_cvd | Charlson indicator cerebrovascular disease |
| charlson_dem | Charlson indicator dementia |
| charlson_cpd | Charlson indicator chronic obstructive pulmonary disease |
| charlson_rhd | Charlson indicator rheumatic heart disease |
| charlson_pud | Charlson indicator peptic ulcer disease |
| charlson_mlivd | Charlson indicator mild liver disease |
| charlson_diab | Charlson indicator diabetes |
| charlson_diabc | Charlson indicator diabetes with complications |
| charlson_plegia | Charlson indicator paralysis (paraplegia) |
| charlson_ren | Charlson indicator renal disease |
| charlson_malign | Charlson indicator malignancy |
| charlson_slivd | Charlson indicator severe liver disease |
| charlson_mst | Charlson indicator Metastatic Cancer |
| charlson_aids | Charlson indicator AIDS |
| hispanic | Hispanic ethnicity |
| census_missing | Census variables observed (0/1) |
| hhld_inc_lt40k | Neighborhood income 40 (<$40k/>=$40k) |
| coll_deg_lt25p | Neighborhood education (<25% college grad/>=25% college grad) |
| phqnumber90 | Total # of Patient Health Questionnaires 9^th^ item (PHQ9s) in prior 90 days |
| phqmode90_0 | Modal PHQ9 value in prior 90 days = 0 |
| phqmode90_1 | Modal PHQ9 value in prior 90 days = 1 |
| phqmode90_2 | Modal PHQ9 value in prior 90 days = 2 |
| phqmax90_0 | Maximum PHQ9 value in prior 90 days = 0 |
| phqmax90_1 | Maximum PHQ9 value in prior 90 days = 1 |
| phqmax90_2 | Maximum PHQ9 value in prior 90 days = 2 |
| phqmax90_3 | Maximum PHQ9 value in prior 90 days = 3 |
| phqnumber183 | Total # of Patient Health Questionnaires (PHQs)_ in prior 183 days |
| phqmode183_0 | Modal PHQ value in prior 183 days = 0 |
| phqmode183_1 | Modal PHQ value in prior 183 days = 1 |
| phqmode183_2 | Modal PHQ value in prior 183 days = 2 |
| phqmax183_0 | Maximum PHQ value in prior 183 days = 0 |
| phqmax183_1 | Maximum PHQ value in prior 183 days = 1 |
| phqmax183_2 | Maximum PHQ value in prior 183 days = 2 |
| phqmax183_3 | Maximum PHQ value in prior 183 days = 3 |
| phqnumber365 | Total # of PHQs in prior 365 days |
| phqmode365_0 | Modal PHQ value in prior 365 days = 0 |
| phqmode365_1 | Modal PHQ value in prior 365 days = 1 |
| phqmode365_2 | Modal PHQ value in prior 365 days = 2 |
| phqmax365_0 | Maximum PHQ value in prior 365 days = 0 |
| phqmax365_1 | Maximum PHQ value in prior 365 days = 1 |
| phqmax365_2 | Maximum PHQ value in prior 365 days = 2 |
| phqmax365_3 | Maximum PHQ value in prior 365 days = 3 |
| dep_dx_pre5y_cumulative | Depression DX, [0,5] years prior |
| anx_dx_pre5y_cumulative | Anxiety DX, [0,5] years prior |
| bip_dx_pre5y_cumulative | Bipolar depression DX, [0,5] years prior |
| sch_dx_pre5y_cumulative | Schizophrenia DX, [0,5] years prior |
| oth_dx_pre5y_cumulative | Other Psychosis DX, [0,5] years prior |
| dem_dx_pre5y_cumulative | Dementia DX, [0,5] years prior |
| add_dx_pre5y_cumulative | ADD DX, [0,5] years prior |
| asd_dx_pre5y_cumulative | ASD DX, [0,5] years prior |
| per_dx_pre5y_cumulative | Personality disorder DX, [0,5] years prior |
| alc_dx_pre5y_cumulative | Alcohol use disorder DX, [0,5] years prior |
| dru_dx_pre5y_cumulative | Drug use disorder DX, [0,5] years prior |
| pts_dx_pre5y_cumulative | PTSD DX, [0,5] years prior |
| eat_dx_pre5y_cumulative | Eating disorder DX, [0,5] years prior |
| tbi_dx_pre5y_cumulative | TBI DX, [0,5] years prior |
| phq8_index_score_calc | PHQ 1^st^-8^th^ items (PHQ8) total score at index visit |
| phq8_missing | PHQ8 missing at index visit (1 if missing) |
| race1 | Race |
| inscat | Insurance type |
| phq9 | PHQ-9 9^th^ item response at index visit |
| unenrolled | individual not enrolled in health system on date of visit |
| First_visit | 1/0. First recorded visit for individual |
| Days_since_prev | Days since previous visit |

**Table S3 (a):** Cross-validated AUC estimates for tuning parameter selection for split-sample prediction model. Highlighted cells indicate the highest cross-validated AUC for all tuning parameter combinations considered.

| Minimum terminal node size | # predictors selected at each split | | |
| --- | --- | --- | --- |
|  | 22 | 11 | 5 |
| 10,000 | 0.796 | 0.806 | 0.793 |
| 25,000 | 0.804 | 0.807 | 0.803 |
| 50,000 | 0.811 | 0.810 | 0.798 |
| 100,000 | 0.805 | 0.805 | 0.809 |
| 150,000 | 0.803 | 0.804 | 0.803 |

**Table S3 (b):** Cross-validated AUC estimates for tuning parameter selection for entire-sample prediction model. Highlighted cells indicate the highest cross-validated AUC for all tuning parameter combinations considered.

| Minimum terminal node size | # predictors selected at each split | | |
| --- | --- | --- | --- |
|  | 22 | 11 | 5 |
| 50,000 | 0.824 | 0.829 | 0.829 |
| 100,000 | 0.825 | 0.832 | 0.827 |
| 250,000 | 0.826 | 0.827 | 0.825 |
| 500,000 | 0.823 | 0.822 | 0.821 |

**Table S4:** Description of development and prospective sample

|  | Development dataset | Prospective validation set |
| --- | --- | --- |
| # visits | 9,610,318 | 3,754,137 |
| # unique people^a^ | 1,078,468 | 601,939 |
| # visits/person, Median (Interquartile range) | 4 (2, 9) | 3 (1,7) |
| # visits with suicide death | 2,318 | 710 |
| Visit-level rate of suicide death (per 100,000 visits) | 24.1 | 18.9 |
| # unique suicide deaths^b^ | 550 | 192 |
| Person-level rate of suicide death (per 100,000 people) | 51.0 | 31.9 |

^a^People may have visits in both the development and prospective validation datasets

^b^Suicide deaths only appear in the development dataset or prospective validation set; there is no overlap.

**Table S5:** Specificity (95% CI) of prediction models from split-sample and entire sample estimation approaches in the development dataset and prospective validation dataset

|  | Split-sample prediction model | | Entire-sample prediction model | | |
| --- | --- | --- | --- | --- | --- |
|  | Testing set, Development | Prospective validation | 5-fold cross-validation, Development | Bootstrap optimism correction, Development | Prospective validation |
| ≥99% | 99.0% (98.9%, 99.1%) | 98.6% (98.6%, 98.7%) | 99.1% (99.0%, 99.1%) | 99.0% (99.0%, 99.0%) | 98.8% (98.7%, 98.9%) |
| ≥95% | 95.1% (95.0%, 95.2%) | 94.0% (93.9%, 94.1%) | 94.9% (94.8%, 95.0% | 95.0% (95.0%, 95.0%) | 94.7% (94.6%, 94.8%) |
| ≥90% | 90.0% (89.9%, 90.2%) | 87.6% (87.5%, 87.8%) | 89.9% (89.7%, 90.0%) | 89.9% (89.9%, 90.0%) | 89.0% (88.8%, 89.1%) |
| ≥75% | 75.0% (74.8%, 75.3%) | 70.4% (70.2%, 70.6%) | 75.0% (74.9%, 75.2%) | 74.9% (74.9%, 75.1%) | 71.9% (71.7%, 72.1%) |

**Figure S1(a):** Moving estimate of cross-validated AUC (95% CI), B=500 bootstrap samples

**Figure S1(b):** Moving estimate of bootstrap optimism-corrected AUC (95% CI), B=500 bootstrap samples

**Figure S2(a):** Moving estimate of cross-validated sensitivity (95% CI) above 99^th^ percentile threshold, B=500 bootstrap samples

**Figure S2(b):** Moving estimate of bootstrap optimism corrected sensitivity (95% CI) above 99^th^ percentile threshold, B=500 bootstrap samples

REFERENCES

1. Ross TR, Ng D, Brown JS, Pardee R, Hornbrook MC, Hart G, et al. The HMO Research Network Virtual Data Warehouse: A public data model to support collaboration. EGEMS (Washington, DC). 2014;2(1):1049.

2. Simon GE, Coleman KJ, Rossom RC, Beck A, Oliver M, Johnson E, et al. Risk of suicide attempt and suicide death following completion of the Patient Health Questionnaire depression module in community practice. J Clin Psychiatry. 2016;77(2):221-7.

3. Simon GE, Johnson E, Lawrence JM, Rossom RC, Ahmedani B, Lynch FL, et al. Predicting suicide attempts and suicide deaths following outpatient visits using electronic health records. Am J Psychiatry. 2018;175(10):951-60.

4. Bakst SS, Braun T, Zucker I, Amitai Z, Shohat T. The accuracy of suicide statistics: are true suicide deaths misclassified? Soc Psychiatry Psychiatr Epidemiol. 2016;51(1):115-23.

5. Cox KL, Nock MK, Biggs QM, Bornemann J, Colpe LJ, Dempsey CL, et al. An examination of potential misclassification of army suicides: results from the Army Study to Assess Risk and Resilience in Servicemembers. Suicide Life Threat Behav. 2017;47(3):257-65.

6. Kroenke K, Spitzer RL, Williams JB. The PHQ-9: validity of a brief depression severity measure. J Gen Intern Med. 2001;16(9):606-13.

7. Breiman L. Random forests. Mach Learn. 2001;45(1):5-32.

8. Breiman L. Some properties of splitting criteria. Mach Learn. 1996;24(1):41-7.

9. Hanley JA, McNeil BJ. A method of comparing the areas under receiver operating characteristic curves derived from the same cases. Radiology. 1983;148(3):839-43.

10. Coley RY, Walker RL, Cruz M, Simon GE, Shortreed SM. Clinical risk prediction models and informative cluster size: Assessing the performance of a suicide risk prediction algorithm. Biom J. 2021.

11. Wright M, Ziegler A. Ranger: A fast implementation of random forests for high dimensional data in C++ and R. J Stat Softw. 2017;77(1):1-17.

12. Belsher BE, Smolenski DJ, Pruitt LD, Bush NE, Beech EH, Workman DE, et al. Prediction Models for Suicide Attempts and Deaths: A Systematic Review and Simulation. JAMA Psychiatry. 2019;76(6):642-51.

13. Simon GE, Shortreed SM, Coley RY. Positive predictive values and potential success of suicide prediction models. JAMA Psychiatry. 2019;76(8):868-9.

14. Steyerberg EW. Clinical prediction models: Springer; 2019.
